# Effect of Different Culture Systems and 3, 5, 3'-Triiodothyronine/Follicle-Stimulating Hormone on Preantral Follicle Development in Mice

**DOI:** 10.1371/journal.pone.0061947

**Published:** 2013-04-15

**Authors:** Cheng Zhang, Xiaoxia Wang, Zhengpin Wang, Wanbao Niu, Baochang Zhu, Guoliang Xia

**Affiliations:** 1 College of Life Science, Capital Normal University, Beijing, Peoples’ Republic of China; 2 State Key Laboratory for Agro-Biotechnology, College of Biological Science, China Agricultural University, Beijing, Peoples’ Republic of China; 3 Animal Science and Technology College, Beijing University of Agriculture, Beijing, Peoples’ Republic of China; Institute of Zoology, Chinese Academy of Sciences, China

## Abstract

The mechanical method to isolate preantral follicle has been reported for many years. However, the culture systems in vitro are still unstable. The aim of this study was to analyze the effect of the culture system of mice preantral follicles on the follicular development in vitro. The results showed that the 96-well plate system was the most effective method for mice follicle development in vitro (volume change: 51.71%; survival rate: 89%, at day 4). Follicle-stimulating hormone (FSH) and Thyroid hormone (TH) are important for normal follicular development and dysregulation of hormones are related with impaired follicular development. To determine the effect of hormone on preantral follicular development, we cultured follicle with hormones in the 96-well plate culture system and found that FSH significantly increased preantral follicular growth on day 4. The FSH-induced growth action was markedly enhanced by T_3_ although T_3_ was ineffective alone. We also demonstrated by QRT-PCR that T_3_ significantly enhanced FSH-induced up-regulation of Xiap mRNA level. Meanwhile, Bad, cell death inducer, was markedly down-regulated by the combination of hormones. Moreover, QRT-PCR results were also consistent with protein regulation which detected by Western Blotting analysis. Taken together, the findings of the present study demonstrate that 96-well plate system is an effective method for preantral follicle development in vitro. Moreover, these results provide insights on the role of thyroid hormone in increasing FSH-induced preantral follicular development, which mediated by up-regulating Xiap and down-regulating Bad.

## Introduction

Follicular development in ovary is highly extremely selective process in mammalian. The destiny of major follicles is atresia. The transition from preantral to antral is the key stage during follicle development, which regulated by survival and death factors [1]. So far, mechanisms of regulating follicle in early stage have not been completely elucidated. In vitro follicle culture is an effective tool to detect the mechanisms of interaction oocyte, granulosa cell and theca cell. Many culture systems of follicle have been developed in different species [2], [3], [4], [5], [6], [7]. Although the fertilizable oocytes have been developed from mouse primordial and early preantral follicles in vitro [8], [9], [10], the follicle culture system for assaying the mechanism of preantral follicular development is still unstable. It is very important to develop FSH (follicle-stimulating hormone) free culture medium in preantral mouse follicle culture, which is used for analyzing the mechanism of factors affecting follicular development. However, the survival rate is only from 8–35% at day 12 of culture [11], [12]. Therefore, many researchers focus on building new culture system. In order to increase follicle survival rate in vitro, FSH is supplied in the culture medium. However, the survival rate after 12 day culture is range from 76.4–99% [9], [13], [14]. Since the culture systems with or without FSH are still unstable, it is necessary to develop new basic culture systems for preantral follicle growth without extra FSH.

FSH is a survival factor to promote follicle development although the preantral-early antral follicle transition is gonadotropin-independent stage [15]. Thyroid hormone (TH (T_3_ and T_4_)] is essential for female reproduction system. Hypo- and hyper-thyroidism are associated with reproductive disorders, including impaired follicular development[16], [17]. The high concentration of T_3_ (100 nM) reduces FSH-stimulated aromatase activity in granulosa cell [18] and impairs mouse preantral follicle development [19]. However, our previous study shows that physiology of T_3_ (1.0 nM) potentiates the granulosa cell survival action of FSH in rat [6]. Whether the combination of hormones exerts promoted action in preantral follicle in mouse is not known.

In our previous study, we also found that T_3_ enhanced FSH-induced granulosa cells survive by up-regulating Xiap (X-linked inhibitor of apoptosis) expression in rat [20]. Xiap is an important regulator of apoptosis through inhibiting caspases 3, 7 and 9 [21], [22], which is highly expressed in healthy follicle [23]. In contrast, Bad (BCL2-associated agonist of cell death) is a cell death regulator, which regulates mitochondrial outer membrane permeabilization and activates the downstream apoptogenic factors. Bad belongs to BCL-2 protein family, the latter is main member in both the initiation and execution of the cell apoptosis [24]. However, whether FSH and or T_3_ regulate Xiap and Bad expression in mouse follicle is still remained unknown.

The preantral follicle culture system in vitro is very important to detect the mechanism of follicular development. In the present study, we cultured preantral follicles (diameter 100–130 µm) in different system and found that 96-well plate system is the most effective one. The follicle survival rate on day 4 was 89% without FSH. In order to examine whether T_3_ and or FSH promote preantral follicular development, we co-cultured preantral follicle with or without hormones. The results showed that FSH alone increased preantral growth, which was enhanced by T_3_, although T_3_ alone was ineffective. Meanwhile, we found that the combination of hormones not only up-regulated Xiap content but also down-regulated Bad expression, which are the explanation of hormones promoted preantral follicle development.

## Materials and Methods

### Materials

All reagents and chemicals used in present study were purchased from Sigma-Aldrich (St. Louis, MO, USA), unless otherwise indicated. α-MEM, Leibovitz L15 medium were from Gibco Bethesda Research Laboratories (GibcoBRL, Carlsbad, Cal,USA). Recombinant human FSH was obtained from the National Hormone and Peptide Program, Harbor-UCLA Medical Center (Torrance, CA). Random decamer primers were from Ambion, Inc. (Austin, TX). Ribonuclease (RNase) inhibitor, RevertAid H Minus M-MULV RT Enzyme, 5 X reaction buffer, and dNTP were from Fermentas International Inc (Qiagen, Valencia, CA). RNeasy Micro kit, deoxyribonuclease I in RNase-free deoxyribonuclease set were purchased from QIAGEN Inc. (Qiagen, Valencia, CA). PCR primers for Xiap, Bad and Rpl19 were from Sanbo China. Rabbit polyclonal antimouse Xiap (2323-PC-050) antibody was purchased from Trevigen Inc. Rabbit polyclonal antimouse Bad (sc-943), monoclonal mouse β-actin antibody, and relative horseradish peroxidase-conjugated second antibodies were purchased from Santa Cruz Biotechnology, Inc. (Santa Cruz, CA, USA).

### Animals

Immature 12 to 14 days old Kunming White female mice (outbreed strain) were purchased from the Beijing Vital Laboratory Animal Technology Co. (Beijing, China). All animal treatment procedures were approved by the Institutional Animal Care and Use Committee of Capital Normal University. Mice were provided with pathogen-free water and food for maintenance and caged in a controlled environment with a 12/12-h light/dark cycle.

### Preantral Follicle Isolation and Culture

Mice were sacrificed by cervical dislocation. Ovaries were removed and placed in 2.5 ml of Leibovitz L15 medium supplemented with 10% FCS, 100 U/ml penicillin and 100 µg/ml streptomycin (all purchased from Life Technologies) [25]. Mechanical dissection of the ovaries was done using a 26- gauge needle. This technique yielded 30–40 good-quality preantral follicles per ovary. In order to minimize the experimental variation during the isolation procedures, the selected preantral follicle was intact with at least one complete granulosa cell layer and a visible, centrally located oocyte for the present studies. The diameter ranged from 100 to 130 µm [26]. The culture medium was composed of a-MEM supplemented with HEPES (10 mM), 0.1% BSA, 3 µg/ml transferrin, 2 ng/ml sodium selenite anhydrous, 5 µg/ml bovine insulin, sodium pyruvate (100 µg/ml), streptomycin (100 µg/ml), and penicillin (100 U/ml) [25].

Selected follicles were rinsed and transferred in culture dish with new media. Then, follicles were randomly divided into 4 groups, and the number of each group was at least 100. The culture systems were modified base on the previous reports. Briefly, the first group was developed in culture dishes (35 mm) with 2 ml medium (Method 1, M1), 1.5 ml sterilized mineral oil were overlaid. In the second group (Method 2, M2), medium was made into 20 µl culture droplet [27], and covered with 3 ml of washed mineral oil. Follicles were placed in the culture droplets, 10 follicles per droplet. Two other groups were cultured in 4-well (Method 3, M3) [28] and 96-well tissue culture plates (Method 4, M4) [12] respectively. For the third group, follicle were cultured in 4-well tissue culture plates, 10 follicles per well with 400 µl culture droplet, which covered with 200 µl of washed mineral oil. In the last groups, follicles were cultured individually in a 96-well plate in 100 µl media. All the follicles were cultured for up to 4 days at 37°C in a humidified atmosphere of 5% CO_2_ in air. Half of the medium was replaced by fresh medium every other day. The diameter of follicle was measured every day as the average of distance between the outer edges of the basement in two perpendicular planes. The results were calculated as followed [25]:

The percentage change of follicular volume (%)  =  (V_n_–V_0_)/V_0_ ×100%.

V_0_ is defined as the volume of follicle at day 0 (the day of follicle isolation); V_n_ means the volume of follicle at day n.

Meanwhile, the oocyte quality was also examined by puncturing follicle. Oocyte death was indicated with particulate matter and accompanied by a progressive darkening and degradation of the follicle. Spontaneous release of a naked oocyte and/or disintegration of the follicle were considered signs of atresia. Follicle survival on day 4 was defined as long as an oocyte remained surrounded by granulosa cells and oocyte was still transparent, round with intact zona pellucid.

In the experiment of detecting the effect of hormones on follicular growth, follicles were cultured individually for up to 4 days in a 96-well plate in 100 µl of α-minimal essential medium (α-MEM) with FSH (10 ng/ml) and/or T_3_ (1.0 nM) [19], [20], [25]. Half of the medium was replaced by fresh medium every other day. The follicle diameter and oocyte quality were also examined as mentioned in the previous part.

### RNA extraction, cDNA synthesis, and real-time PCR analysis

Total RNA of mouse follicle were extracted from the above indicated groups using RNeasy Micro kit according to the manufacturer’s instructions [26]. Then, reverse transcription (RT) was conducted as follow: 0.2 µg total RNAs were reverse transcribed in a final volume of 20 µl solution containing 4 µl 5×reaction buffer, 2 µl 10 mM dNTP, 20 U of RNase inhibitor, 200 U RevertAid H Minus M-MULV RT enzyme, random decamer primers, and RNase free H_2_O [29]. Real-time PCR was then conducted to quantify gene mRNA levels using an ABI 7500 real-time PCR instrument (Applied Biosystems, Foster City, CA). PCR primer sequences for Xiap were 5′-TCACAGCACTCCAACTCTAATC-3′ (forward) and 5′- GACCTTCCGAGTGACCATTT-3′ (reverse). The Bad primer sequences were 5′- AGGATGAGCGATGAGTTTGAG-3′ (5′ forward primer) and 5′- TCCCACCAGGACTGGATAA-3′ (3′ reverse primer). And *Rpl19* sense and antisense PCR primers were 5′-CTGAAGGTCAAAGGGAATGTGTTC-3′ and 5′-TGGTCAGCCAGGAGCTTCTTG-3′, respectively. Amplification reaction was then performed using the QuantiTect SYBR Green PCR kit. To avoid false positive signals, dissociation-curve analyses were performed at the end of the amplification and the PCR products were run on a 1.5% agarose gel to confirm the sizes. Moreover, the PCR products were purified and sequenced to verify sequence identity. A standard curve was included for each gene, and in each PCR, the target gene mRNA abundance was quantified by using the standard curve and was expressed as a ratio to ribosomal protein L19 (*Rpl19*) values, a housekeeping gene, by the 2-ΔΔCt method [30], and then expressed as fold of 0 h respectively before statistical analysis. PCR without reverse-transcribed cDNA were used as negative controls.

### Western Blotting

Western blot analysis was performed as described previously [14], [26], [29]. Briefly, follicles were collected after treatment. Protease inhibitors PMSF (10 µm), aprotinin (50 ug/ml) and sodium orthovanadate (1 mM) were added to buffers and follicular protein extracted. All the procedures were carried out on ice. Cell pellets were homogenized by sonication and centrifuged (14,000 x g, 4C, 30 min). Aliquots of proteins (15 µg) were resolved by SDS-PAGE (10%) and electrotransferred to nitrocellulose membranes (Protran; Schleicher Schuell UK Ltd, London). Membranes were blocked (RT, 1 h) in TBST [Tris-buffered saline (pH 8.0) with 0.05% Tween 20 (TBS-T), 5% dehydrated nonfat milk powder]. And then, membranes were incubated (4°C, overnight) with blotto containing 0.1 g/ml Xiap (1∶1000), Bad (1∶1000), β-actin (1∶10,000) antibody. Then washed in TBS-T (3×5 min), incubated in HRP-conjugated secondary antibody (1∶3000, 1∶3000, 1∶5000, respectively) in blotto, and washed again in TBS-T. Peroxidase activity was visualized with the ECL kit according to the manufacturer's instructions, and protein content was determined by densitometrically scanning the exposed x-ray film.

### Statistical Analysis

All experiments were performed at least three times. Results were presented as means ± SEM of at least three independent experiments, as detailed in the figure legends. For multiple-group analysis, one- or three-way ANOVA and the Bonferroni post test were used. Statistical significance was defined as P<0.05.

## Results

### Comparison of effects of different culture system

**Figure 1:**
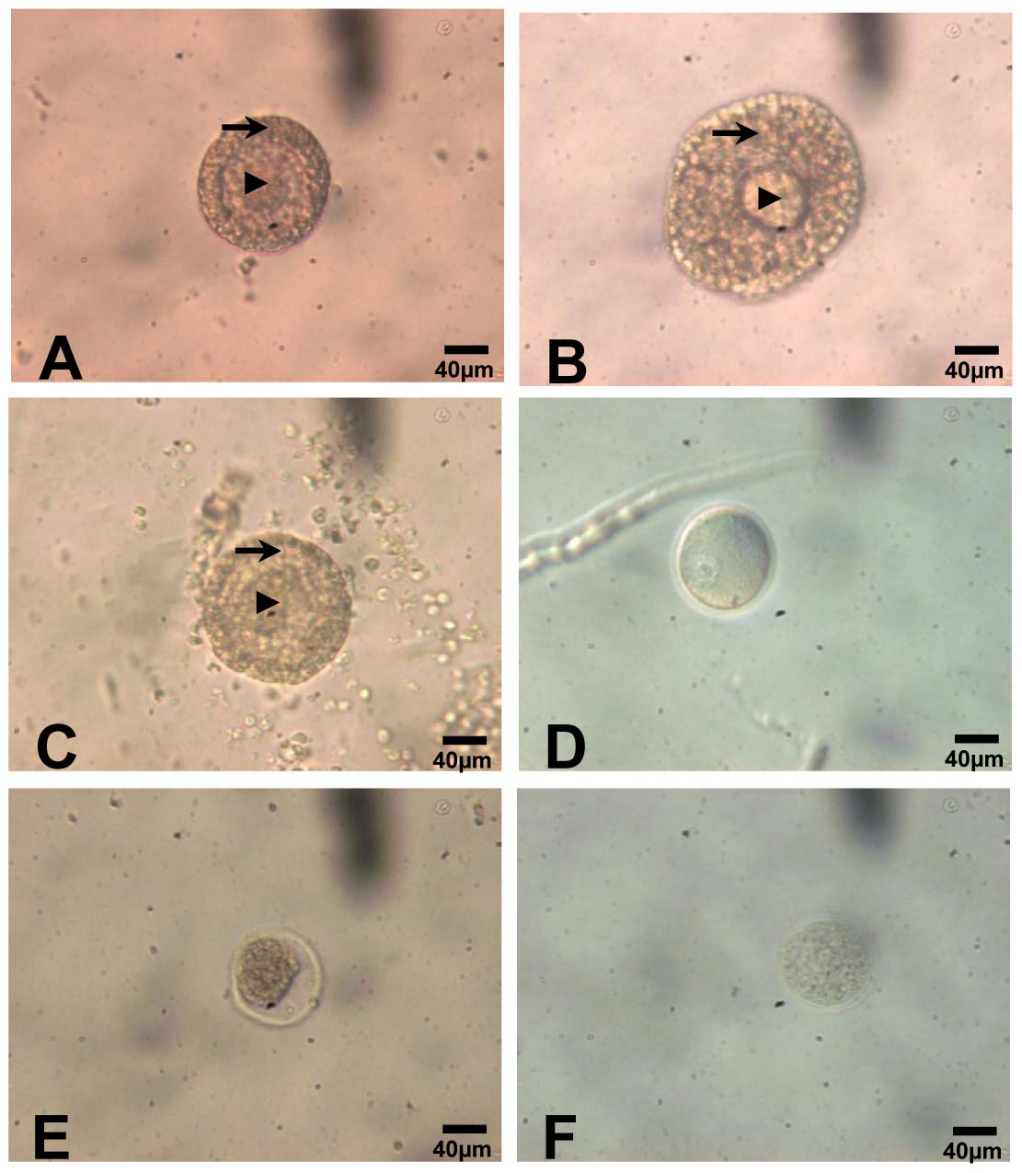
Representative examples of preantral follicle and oocyte in different phase. (A) Follicle isolated mechanically (day 0): central germinal vesicle (GV)-stage oocyte (arrow head) with a thin zona pellucida, surrounded by two layers of cuboidal granulosa cells (arrow), a basal membrane and a single layer of theca cells (original magnification ×200). **(B)** Preantral follicle after 4 days of culture with FSH (10 ng/ml) had intact basal membrane with proliferated granulosa cells (original magnification ×200). **(C)** Preantral follicle after 4 days of culture had shrunk with smaller oocyte (original magnification ×200). **(D)** The follicle culture on day 4 was punctured and healthy oocyte was released. **(E, F)** The oocytes from those darken and or shrunk follicles showed retrogression with many particles or big gap between oocyte and zona pellucida (original magnification ×200). Bars  =  40 µm.

**Figure 2:**
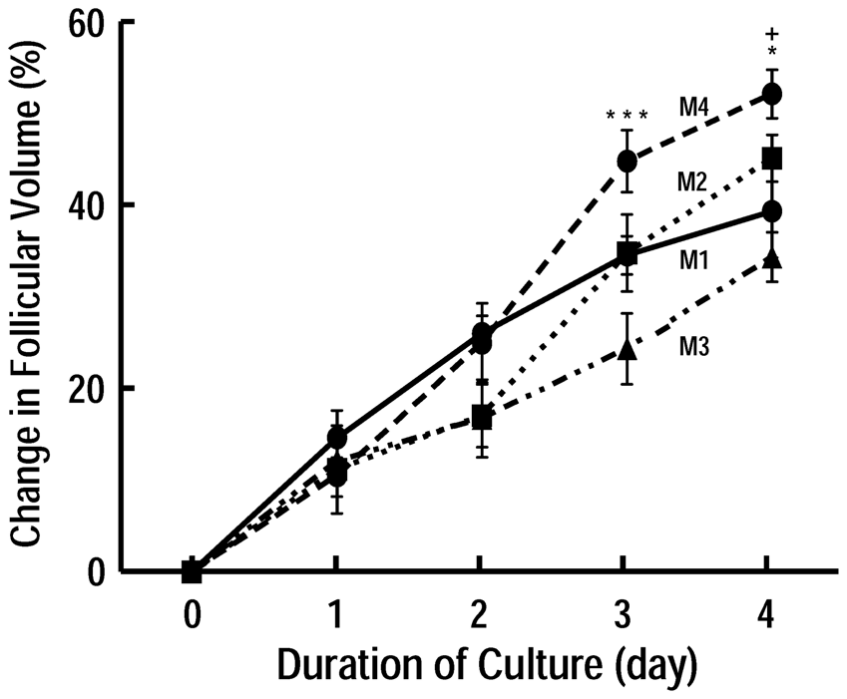
Effect of different culture system on preantral follicular development *in vitro.* Mouse preantral follicles (100–130 µm in diameter) were isolated mechanically and cultured in different systems. M1 group was developed in culture dishes (35 mm) with 2 ml medium. In the second group (Method 2, M2), medium was made into 10×20 µl culture droplet, and covered with 3 ml of washed mineral oil. Follicles were placed in the culture droplets, 10 follicles per droplet. Two other groups were cultured in 4-well (Method 3, M3) and 96-well tissue culture plates (Method 4, M4) respectively. Follicular diameter was measured daily and results were expressed as change in follicular volume. Note that follicles grew faster in M4 group than M1 and M3 groups although the statistic analysis was not significant among others groups. ^+^P<0.05 vs. M1 at the same culture duration, *P<0.05, ***P<0.001, vs. M3 at the same culture duration.

Preantral follicles used for experiment were manually isolated and cultured in four culture systems for 4 days. A representative follicle is shown in Figure 1-A, B. The results showed that there were no significant differences among 4 groups at Day 2. Follicles after 3 days of culture in M4 group (44.42±0.76%) grew faster than those in M3 (24.16±0.82%) groups at the same duration (P<0.001). Although follicles cultured in M1, M2 and M3 showed minimal growth (follicular volume change at culture day 4: 39.02±0.41%, 44.74±0.25%, 34.07±0.38%, respectively Fig. 2), no significant difference in follicular growth were observed among three groups (P>0.05). However, follicles cultured in 96-well plate (M4) significantly grew faster than other groups at culture day 4 (51.71±0.46%, P<0.05, Figure 2) except M2 group. At the end of follicular culture, follicles were punctured and oocytes were released. A representative health oocyte is shown in Figure 1-D. The survival rate of follicle was calculated. The results showed that the M4 group presented a significantly higher rate of healthy oocyte compared with M1, M2, and M3 respectively. Meanwhile, the percentages of healthy oocyte were not statistically differences among others groups (Table 1).

**Table 1:** Effect of different culture system on the oocyte growth of preantral follicles at day 4.

		No. of follicles	
Culture condition	Follicle diameter at start (means ± SEM)	Plated on day 0	Surviving on day 4 (%)
M1	118.40±3.52	120	55^a^
M2	115.92±4.21	130	63^a^
M3	117.86±5.61	115	71^a^
M4	119.29±3.93	120	89^b^

a,b Means within a column with different superscripts differ (P<0.05)

### Effect of hormones on follicular growth in vitro

**Figure 3:**
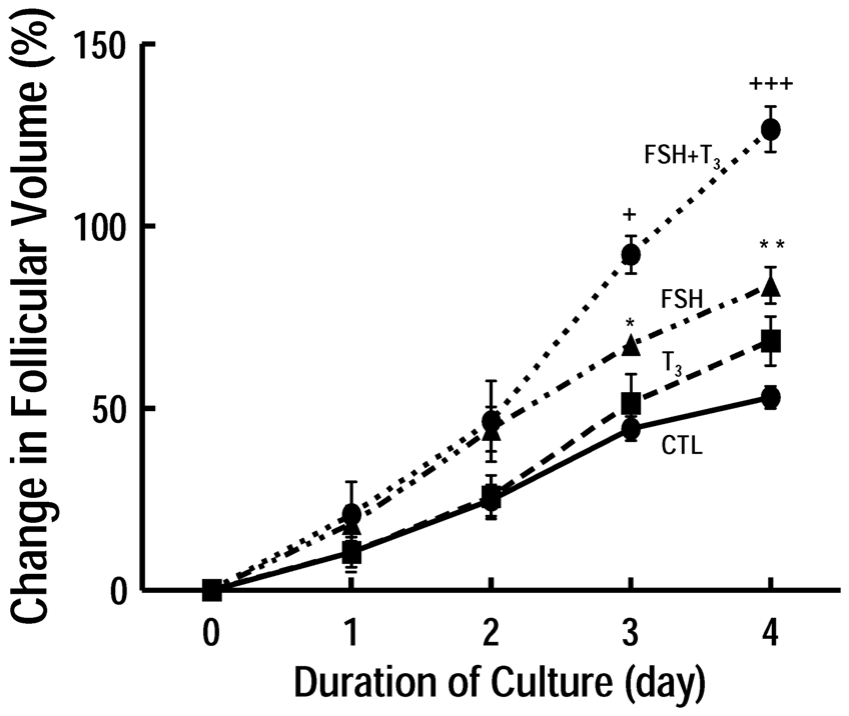
Effect of FSH and T_3_ on preantral follicular growth *in vitro.* Preantral follicles were cultured for 4 days with or without FSH (10 ng/ml) and T_3_ (1.0 nM). Follicles cultured in the absence of FSH and T_3_ was named as CTL group. Follicular diameter was measured daily and results were expressed as change in follicular volume and represented as means ± SEM of a total of 120 follicles from four independent experiments. Note that T_3_, while ineffective alone, significantly increased FSH-induced preantral follicular growth at Day 4 (P<0.001). ^+^P<0.05, ^+++^P<0.001 vs. FSH alone at the same culture duration, *P<0.05, **P<0.01, vs. CTL at the same culture duration (Three way ANOVA).

In order to evaluate the effect of FSH and or T_3_ on preantral follicle development, follicles were co-treatment with hormones for 4 days duration. The follicular volume change curve during the 4-day culture period is presented in Figure 3. In the control group (Preantral follicles cultured in the absence of FSH and T_3_; CTL), follicles were not significantly increased in volume after 4 days of culture. There were no significant difference between T_3_-treated (63.41±3.97%) and CTL (53.02±1.09%) groups (P>0.05) in follicular growth at day 4 of culture. However, FSH alone was statistically different compared with CTL groups from day 3 (67.49±1.13 vs. 44.42±1.08%, P<0.05, at day3; 83.73±1.27 vs. 53.02±1.37%, P<0.01, at day4, vs. CTL). Moreover, T_3_ synergized with FSH in inducing preantral follicular growth although T_3_ alone was ineffective (126.61±3.14%, P<0.001 vs. FSH alone) after 4 days culture. Furthermore, the enhancement of T_3_ on FSH-induced follicular growth was evident as early as Day 3 (92.15±2.18% P<0.05 vs. FSH alone).

### Effect of FSH and T_3_ on Xiap expression in vitro

**Figure 4:**
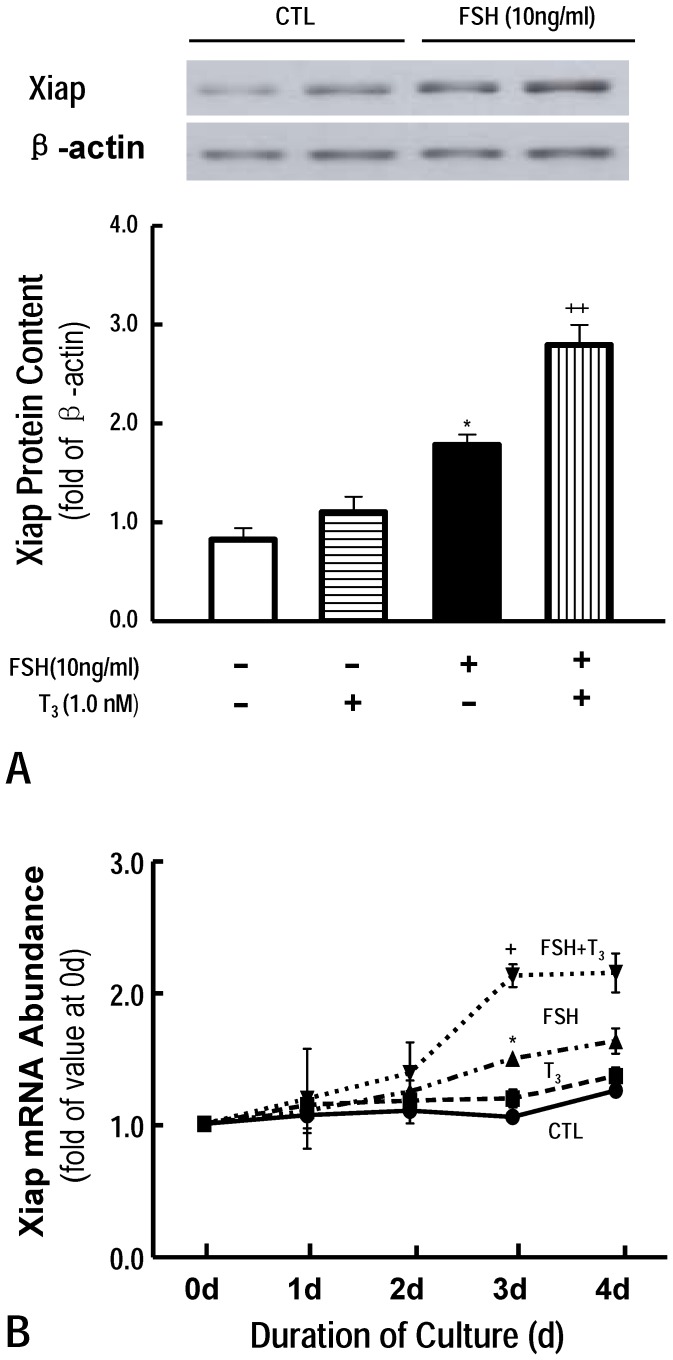
Effect of FSH and T_3_ on follicular Xiap expression *in vitro*. Results are represented as means±SEM of four independent experiments. Note that T_3_, although ineffective alone, significantly increased FSH-induced Xiap expression (T_3_ 1.0 nM; P<0.01). *: P<0.05 vs CTL. +: P<0.05, ++: P<0.01vs FSH alone (Two way ANOVA)

Xiap is an anti-apoptotic factor, which is regulated by gonadotropin in granulosa cells [23], [31]. In the present study, we used follicular cultured system in vitro to detect whether FSH and or T_3_ regulated Xiap expression. Xiap mRNA content was detected by QRT-PCR. The results showed that Xiap mRNA content was significantly up-regulated by FSH compared with that in CTL groups [1.49±0.13 (FSH) vs. 1.05±0.07 (CTL), P<0.05, Figure 4]. The level of Xiap mRNA was gradually increased during the culture period in the FSH treatment. The up-regulation of FSH on Xiap gene expression was also confirmed by western blotting [1.79±0.13 (FSH) vs. 0.83±0.14 (CTL), P<0.05, Figure 4]. Although T_3_ alone was ineffective, T_3_ significantly enhanced FSH effect on regulating Xiap expression [2.13±0.32 (mRNA), 2.78±0.19 (protein), P<0.01, Figure 4].

### Effect of FSH and T_3_ on Bad content in vitro

**Figure 5:**
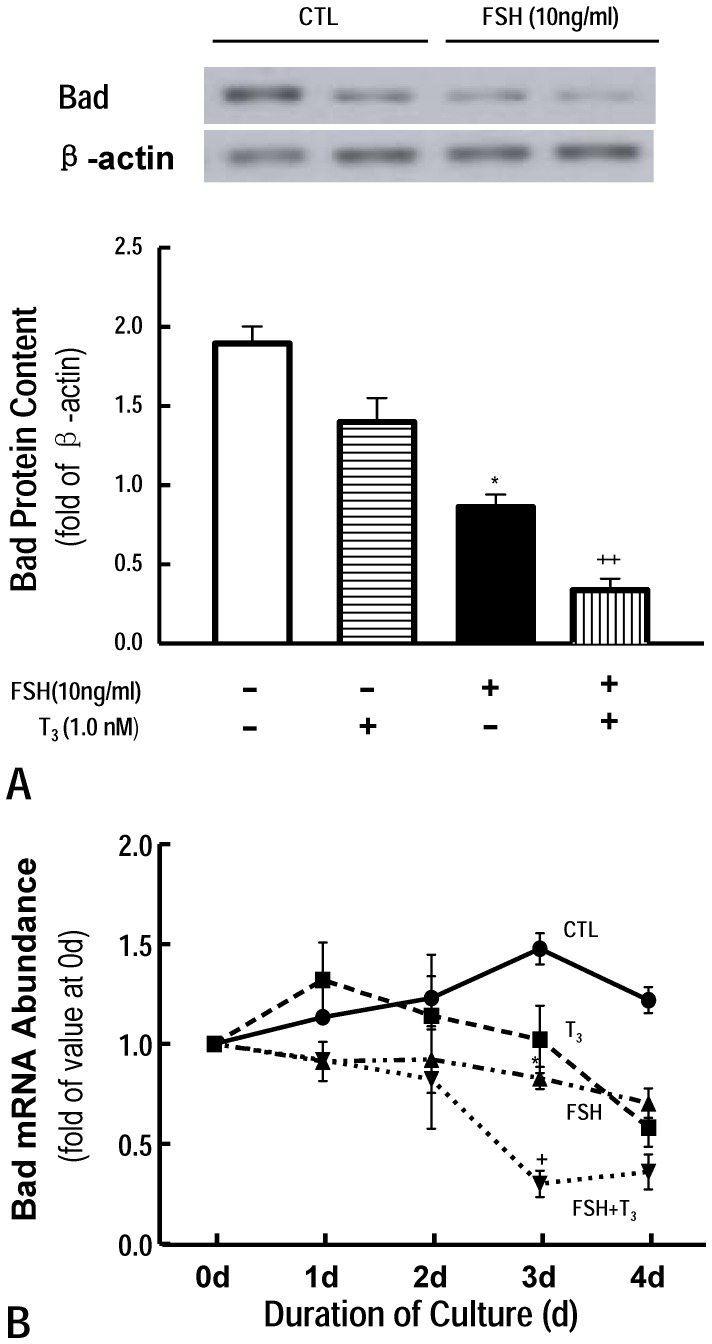
Effect of FSH and T_3_ on follicular Bad expression *in vitro*. Results were represented as means±SEM of four independent experiments. FSH significantly down-regualted Bad expression (P<0.05), the down-regulation effect was dramatically enhanced by T_3_ (P<0.01) although T_3_ alone was ineffective. *: P<0.05 vs CTL; +, P<0.05; ++, P<0.01 vs FSH (Two way ANOVA).

It is reported that Bad is a member of pro-apoptotic Bcl-2 family, which are triggered by intracellular signals such as by cellular or DNA damage via intrinsic/mitochondrial apoptosis pathways[32], [33]. In order to determine the effect of hormones on regulating Bad expression, QRT-PCR was also used to detect Bad mRNA content. As shown in Figure 5, FSH alone (0.83±0.09) significantly decreased the level of mRNA compared with CTL group (1.48±0.11) at day 3, which was dramatically enhanced by T_3_ (0.31±0.08, FSH+T_3_). Interestingly, T_3_ alone also significantly down-regulated the abundance of Bad mRNA at day 4 [0.58±0.12 (T_3_) vs. 1.22±0.08 (CTL), P<0.05, Figure 5]. The effect of hormones on the Bad protein in follicle was determined by western blotting. The protein content of Bad in follicles was not obvious changed in T_3_ group (1.40±0.18) compared with that in CTL group (1.89±0.13) although T_3_ down-regulated Bad mRNA level. However, T_3_ dramatically enhanced the down-regulation effect of FSH on Bad protein content [0.34±0.10 (FSH+T_3_) vs. 0.86±0.09 (FSH), P<0.01, Figure 5].

## Discussion

In the present study, four methods had been used in attempts to develop culture systems for preantral follicles. Although the volume change was apparently affected after 4 days culture, results are still preliminary for us to make hard statements on follicular development. Therefore, we checked the oocyte morphology at the end of culture period. The results were consistent with volume change.

The advantage of M1, M2 and M3 system was that the crosstalk between follicles was still kept although the follicles grew slower than those in M4. However, it was hard to track the follicle growth individually. Meanwhile, there were some atresia follicles in the first three culture systems, which may secreted cell death inducers and latter impacted other follicles in the whole community [34]. Maybe this is the reasonable explanation for the death rate was higher than that in 96-well plate system (M4). In addition, all the culture systems were with mineral oil overlay except M4. Since the mineral oil extracts steroid hormones (Estrogen and Progesterone) and sterols from the culture medium [35], [36] or by impurities that cause toxic effects (such as ammonium) [37], [38], [39], [40], [41], which may possibly be explained that different vehicles with the same medium caused different survival rate.

For 96-well plate method follicles are individually cultured in the well and have the advantagement of measuring diameter and oocyte growth without disturbing situation from other follicles. The culture condition provides effective method to track the follicular growth in the whole culture duration although the crosstalk among follicles are cut off [12]. In this system, the normal three-dimensional structure of follicle is still kept. The survival rate in the FSH free medium was 89%, which is similar with previous reports in the medium with FSH [9], [13], [14]. Although the culture duration in the present study is only 4 days, it is an effective and stable system for further research. And then, this system was used to evaluate the effects of hormones on follicular development.

As a positive factor, FSH is an dominant factor for follicle development and suppresses atresia by inhibiting apoptosis [42], [43]. Moreover, many reports show that dysregulation of TH level is related with reproductive disorders [16], [44], [45]. The additional presence of TH significantly improve ovarian condition in hypothyroidism which is markedly hampered the follicle development [46]. FSH is an essential factor for follicle growth in vitro and we demonstrated that mice preantral follicles developed and responded to FSH in a 4-day culture system in our study. In the FSH only culture condition, granulosa cell proliferation was obvious. It has been showed that FSH receptors are detected on granulosa cells from the earlier preantral follicle [47]. FSH plays an important role in promoting follicular development as a dominant inducer of granulosa cell proliferation and differentiation [48]. When both hormones (FSH + T_3_) were present in the culture media, follicle growth was significantly faster than FSH alone group in volume change although T_3_ alone was ineffective. Moreover, the effect of T_3_ was biphasic and within the physiological range of circulating levels of T_3_ [19]. Further in-vitro development was also evaluated by examining oocyte quality. The pattern of oocyte healthy rate was also showed highest in the group of hormone combination.

The previous results revealed that the presence of hormones in culture medium is necessary to promote preantral follicular development. However, the mechanism that thyroid hormone synergizes with FSH in the development of preantral follicle is still unknown. The transition from preantral-early to antral follicle is the key stage of follicular development, which is most susceptible to follicular atresia [49]. The survival of follicles in each reproductive cycle may occur through up-regulating the survival factors and/or removal of the cell death inducers by an appropriate stimulator.

Xiap is the most potent suppressor of mammalian cell apoptosis and necrosis through the direct binding with caspase-3, -7 and -9 via their BIR2 or BIR domains [50], which regulates the cell cycle and inflammation[31], [51]. Xiap is also an anti-apoptotic factor in ovarian cells, which is up-regulated by gonadotropin during follicular development [51]. In the present study, T_3_ enhanced FSH-induced Xiap protein up-regulation although T_3_ alone had no effect on Xiap content. The latter regulation maybe mediated by increasing transcription level and or stabilization of the message since the content of Xiap mRNA was also increased by the co-treatment of FSH and T_3_.

Xiap has been shown to modulate the Bax/cytochrome *c* pathway by inhibiting caspase-9 [51]. It is well established that Bad belongs to Bcl-2 family proteins [32]. Bad is a cell death inducer that constitutes a critical control point in the intrinsic apoptosis pathway, which occurs the releasing of cytochrome *c* from mitochondria and the subsequent activation of downstream apoptogenic factors [52], [53]. The present studies showed that both Bad protein and mRNA levels were down-regulated by co-treatment of FSH and T_3_, although T_3_ alone had no any significant effect in protein regulation. Whether the decrease in Bad mRNA abundance by T_3_ alone is due to decreased gene transcription and unstabilization of the message, remains to be determined. These results suggest that down-regulation of Bad also plays an important role in the protective effect of hormones on the follicular development by the intrinsic apoptosis pathway. It is well known that follicle contains theca cell, granulosa cell and oocyte. Although the change of genes expression were regulated by hormones in the present study, which type cell is the dominant cell regulated by hormones remains investigated.

Our observations suggest that the role of FSH and T_3_ in the regulation of follicular development is mediated by up-regulating Xiap expression and down-regulating Bad content simultaneously. It is well established that the crosstalk among theca cell-granulosa cell-oocyte is important for follicle development and oocyte maturation. In addition, autocrine factors maybe also involved the follicular growth, which are regulated by FSH and or T_3_.

In conclusion, the results of the present study indicate that 96-well plate culture system is the most effective method for preantral follicular culture in vitro and T_3_ potentiates follicular development action of FSH through up-regulation of Xiap and down-regulation of Bad. However, whether the effect of hormones is follicular stage-dependent is not known. Moreover, the precise mechanism of hormones on promoting follicle development need investigated in the future for better understanding the action of hormones in regulating follicular fate.
